# Reanalysis of exome negative patients with rare disease: a pragmatic workflow for diagnostic applications

**DOI:** 10.1186/s13073-022-01069-z

**Published:** 2022-06-17

**Authors:** Gaby Schobers, Jolanda H. Schieving, Helger G. Yntema, Maartje Pennings, Rolph Pfundt, Ronny Derks, Tom Hofste, Ilse de Wijs, Nienke Wieskamp, Simone van den Heuvel, Jordi Corominas Galbany, Christian Gilissen, Marcel Nelen, Han G. Brunner, Tjitske Kleefstra, Erik-Jan Kamsteeg, Michèl A. A. P. Willemsen, Lisenka E. L. M. Vissers

**Affiliations:** 1 Department of Human Genetics, Radboud University Medical Center, Nijmegen, The Netherlands; 2 Donders Institute for Brain, Cognition and Behaviour, Nijmegen, The Netherlands; 3 Department of Pediatric Neurology, Radboud University Medical Center, Nijmegen, The Netherlands; 4 Radboud Institute for Molecular Life Sciences, Nijmegen, The Netherlands; 5 Department of Clinical Genetics, Maastricht University Medical Centre+, Maastricht, The Netherlands

**Keywords:** NGS-based resequencing, Systematic reanalysis, Rare disease, Diagnostic implementation of recommended ACMG guideline, Longitudinal follow-up of systematic cohort

## Abstract

**Background:**

Approximately two third of patients with a rare genetic disease remain undiagnosed after exome sequencing (ES). As part of our post-test counseling procedures, patients without a conclusive diagnosis are advised to recontact their referring clinician to discuss new diagnostic opportunities in due time. We performed a systematic study of genetically undiagnosed patients 5 years after their initial negative ES report to determine the efficiency of diverse reanalysis strategies.

**Methods:**

We revisited a cohort of 150 pediatric neurology patients originally enrolled at Radboud University Medical Center, of whom 103 initially remained genetically undiagnosed. We monitored uptake of physician-initiated routine clinical and/or genetic re-evaluation (ad hoc re-evaluation) and performed systematic reanalysis, including ES-based resequencing, of all genetically undiagnosed patients (systematic re-evaluation).

**Results:**

Ad hoc re-evaluation was initiated for 45 of 103 patients and yielded 18 diagnoses (including 1 non-genetic). Subsequent systematic re-evaluation identified another 14 diagnoses, increasing the diagnostic yield in our cohort from 31% (47/150) to 53% (79/150). New genetic diagnoses were established by reclassification of previously identified variants (10%, 3/31), reanalysis with enhanced bioinformatic pipelines (19%, 6/31), improved coverage after resequencing (29%, 9/31), and new disease-gene associations (42%, 13/31). Crucially, our systematic study also showed that 11 of the 14 further conclusive genetic diagnoses were made in patients without a genetic diagnosis that did not recontact their referring clinician.

**Conclusions:**

We find that upon re-evaluation of undiagnosed patients, both reanalysis of existing ES data as well as resequencing strategies are needed to identify additional genetic diagnoses. Importantly, not all patients are routinely re-evaluated in clinical care, prolonging their diagnostic trajectory, unless systematic reanalysis is facilitated. We have translated our observations into considerations for systematic and ad hoc reanalysis in routine genetic care.

**Supplementary Information:**

The online version contains supplementary material available at 10.1186/s13073-022-01069-z.

## Background

Exome sequencing (ES) is a genetic diagnostic approach used to reduce the diagnostic odyssey of patients and in particular in children with complex neurological disorders of presumed genetic origin [1, 2]. Understanding of the genetic defect may provide information on prognosis, improve patient management, guide therapeutic choices, and allow for more informed reproductive options for family members [3]. In addition, a diagnosis often facilitates access to supportive care systems [4–7].

As part of post-test counseling procedures, patients without a conclusive diagnosis are advised to recontact their referring clinician to discuss new diagnostic opportunities in due time. A number of studies in pediatric neurology have shown a diagnostic yield of ES of ~ 30% [1, 2, 8], but this is likely to increase with time through new developments. Improved enrichment technologies, optimized sequence chemistry, and more sophisticated analytical tools continuously allow the discovery of previously unrecognized clinically relevant variants [9]. In addition, systematic re-evaluation of existing ES datasets allows reinterpretation and novel diagnoses because of new disease-gene associations unknown at the time of initial analysis. Estimates suggest that the diagnostic yield by ES could be increased with ~ 15%, when using up-to-date software, literature, and phenotypic information for reinterpretation [3, 10–15]. However, previous systematic studies have limited their selves to reanalysis of existing data at the time of their research, disregarding the effects of reanalysis in a patient initiated diagnostic workflow. Moreover, despite ongoing technological developments for next generation sequencing (NGS), potentially providing additional diagnosis, models on clinical reanalysis are limited to the reuse of existing data and rarely include resequencing [13, 14, 16], despite recommendations of the American College of Medical Genetics (ACMG) [17].

To emphasize the significance of ES reanalysis, we revisited a cohort of 150 children with a complex neurological disorder, originally enrolled at Radboud University Medical Center for a clinical utility study on the performance of ES, which was shown to be representative for the broad phenotypic spectrum of disorders seen in the routine pediatric neurology diagnostic trajectory [1]. To gain insight into the relative contribution of reanalysis strategies of ES, we monitored the increase of diagnostic yield over a 5-year period resulting from routine care, based on patients that indeed recontacted their referring clinician. Moreover, we subsequently performed a systematic reanalysis, including resequencing with an advanced clinical ES pipeline (i.e., including low quality variants, copy number variant (CNV) analysis, and up-to-date disease-gene panels), of all patients in this cohort without a genetic diagnosis, with the patients that did not initiate reanalysis, allowing to translate the findings into a generalizable and effective strategy for clinical reanalysis.

## Methods

### Clinical cohort

In this study, we revisited the 103 genetically undiagnosed patients from an original cohort of 150 consecutive patients with complex neurological symptoms of suspected genetic origin who were seen at the department of pediatric neurology at the Radboud University Medical Center [1]. Patients (and their unaffected parents) were included between November 2011 and January 2015 [1]. The original study was approved by the Medical Ethics Review Committee Arnhem-Nijmegen under 2011/188 and the systematic evaluation of diagnostic follow-up and innovation under 2020-7142.

### Systematic ES reanalysis

A three-step process was used to examine the increase of the diagnostic yield within the original pediatric neurology cohort of 150 patients [1]. In brief, we first retrospectively collected all genetic diagnostic tests that were performed after the original data freeze of July 2015. Physician-initiated analyses (ad hoc analyses) consisted of reanalysis of existing exome data or analysis of newly generated exome data (i.e., resequencing). Updated pipelines for variant calling allowed CNV analysis as well as the analysis of variants that previously failed quality control (QC) parameters, e.g., total number of reads or percentage of variant reads. Bioinformatic methods were as described before; in short, sequence reads were aligned to the hg19 reference genome using BWA, CNVs were called by CoNIFER [18], and SNVs/indels were called by the GATK unified genotyper [19]. Reannotations reflected gene-panel updates and the Variant Effect Predictor (VEP) [20] for prioritization as well as knowledge bases such as gnomAD [21], in-house variant frequencies, OMIM phenotypes, and new literature searches. Reinterpretation of previous class 3 variants/variants of uncertain clinical significance (VUS) included literature studies, segregation analysis, and/or functional follow-up.

Second, for all patients who did not have genetic diagnostic follow-up since 2015, we performed systematic reanalysis of the original ES data using the updated pipelines and knowledge databases provided that the original data was compatible with current diagnostic bioinformatic pipelines [18, 19].

Third, for patients who remained undiagnosed after reanalysis under steps one and two, we performed resequencing following diagnostic procedures [1], using Twist Bioscience Human Core Exome+ RefSeq Panel Enrichment Kit (TWIST Bioscience, San Francisco, CA, USA) and the Illumina NovaSeq 6000 at 100x coverage (Illumina, San Diego, CA, USA).

### Variant classification

Variant interpretation of ES (re)analyses were performed as follows. First, a disease-gene panel strategy was performed by in silico enrichment of single-nucleotide and copy-number variants (SNVs and CNVs) in genes with established disease-associations related to the patients phenotype [1]. Subsequently, (de novo) variants (SNVs, indels and CNVs) outside these gene-panels were evaluated for pathogenicity, as well as their disease relevance (i.e., open exome strategy). Prioritization of the variants was based on conservation and predicted impact using the VEP [20] and gnomAD [21]. For classification of SNVs and indels, we used a classification based on the guidelines jointly established by the Association for Clinical Genetic Science (ACGS) and the Dutch Society of Clinical Genetic Laboratory Specialists (VKGL) [22]: (1) benign/likely benign, (2) variant of uncertain significance (VUS), or (3) likely pathogenic/pathogenic. CNVs were classified according to the European guidelines for constitutional cytogenomic analysis (class 1 to class 5) [23].

All remaining VUS were once more reassessed in February 2021. Finally, the outcomes of the ES analyses were described according to the categories in the original study: (1) no diagnosis (e.g., absence of variants explaining disease phenotype), (2) a possible diagnosis (VUS in known disease gene related to patients phenotype or a (likely) pathogenic variant in a candidate disease gene), or (3) a conclusive diagnosis ((likely) pathogenic variant explaining the patients phenotype) [1].

## Results

**Fig. 1:**
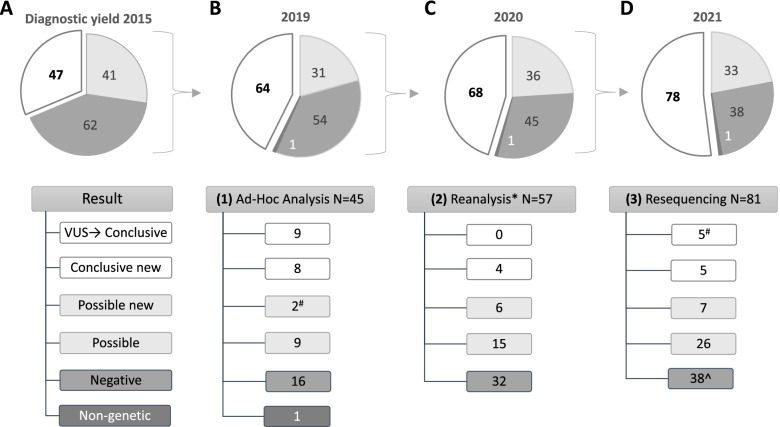
Three-step reanalysis strategy of clinical exome data reanalysis. Schematic representation of the evolution of the diagnostic yield in our cohort of 150 patient seen in pediatric neurology. The initial diagnostic yield is presented in **A** and follows the three steps of reanalysis that led to new diagnoses. Step 1 (**B**) involved the continued diagnostic odyssey in the routine care trajectory. Step 2 (**C**) involved the reanalysis of available exome data when data was suitable*, and step 3 (**D**) included the systematic resequencing and reanalysis for the remaining unsolved cases. Of note, two diagnoses were made by reclassification of variants of unknown significance (VUS) detected in the ad hoc analysis^#^, and two VUS were rejected based on population frequency^

Previously, we identified genetic diagnoses in 47 of 150 patients with disorders of presumed genetic origin, by studying the clinical utility of exome sequencing [1]. From 103 patients without a genetic diagnosis in 2015 (Fig. 1A), 45 revisited our clinics and received additional ad hoc diagnostic testing as well as clinical reanalysis by a pediatric neurologist. For one of the patients, MRI determined that the origin of disease was an acquired cerebral palsy rather than a disorder of genetic origin. The other 44 patients had reanalysis of existing exome data (*n* = 29) or resequencing followed by reanalysis (*n* = 15, Additional file 1: Table S1). This yielded 17 new conclusive genetic diagnoses. (Table 1—step 1, Fig. 1B).

**Table 1:** The evolution of diagnostic yield; 31 novel genetic diagnoses after 5 years of systematic follow-up

Patient ID^**a**^	Gender		Variant in VCF 2015	Gene in panel 2015		This study		Initial analysis 2015^**a**^	
		Gene			Step	Identified by	Note	Result	Gene
**6**	F	**HNRNPK**	Yes	No	1	Variant reclassification (new disease-gene)	OMIM: 616580	Possible	HNRNPK
**32**	F	**CSNK2A1**	Yes	No	1	Variant reclassification (new disease-gene)	OMIM: 617062	Possible	CSNK2A1
**40**	F	**ADPRHL2**	Yes	No	1	Variant reclassification (new disease-gene)	OMIM: 618170	Possible	ADPRHL2
**46**	M	**DHX30**	Yes	No	1	Variant reclassification (new disease-gene)	OMIM: 617804	Possible	DHX30
**48**	M	**CSNK2B**	Yes	No	3	Variant reclassification (new disease-gene)	OMIM: 618732	Possible	CSNK2B
**84**	M	**c19orf12**	Yes	No	1	Variant reclassification (new disease-gene)	OMIM: 614298	Possible	C19orf12
**104**	F	**PPP2CA**	Yes	No	1	Variant reclassification (new disease-gene)	OMIM: 618354	Possible	PPP2CA
**109**	M	**KAT8**	Yes	No	3	Variant reclassification (new disease-gene)	OMIM: 618974	Possible	KAT8
**112**	M	**SNORD118**	Yes	No	1	Variant reclassification (new disease-gene)	OMIM: 614561	Possible	SNORD118
**93**	M	**TBC1D24**	Yes	Yes	3	Variant reclassification (additional disease-gene)	PMID: 31257402	Possible	TBC1D24
**34**	M	**HSD17B10**	Yes	Yes	1	Variant reclassification (additional testing)	Metabolic investigation	Possible	HSD17B1
**103**	M	**ACSL4**	Yes	Yes	1	Variant reclassification (additional testing)	Mother de novo	Possible	ACSL4
**147**	F	**PIK3R2**	Yes	Yes	1	Reanalysis (additional testing)	Segregation in affected brother	Possible (other)	DHCR24
**138**	M	**NAA15**	Yes	No	1	Update pipeline (gene panel-new)	OMIM: 617787	No cause	–
**129**	F	**SRCAP**	Yes	Yes	3	Update pipeline (gene panel-additional); variant reclassification (additional disease-gene)	PMID: 33909990	No cause	–
**113**	M	**NALCN**	Yes	Yes	2	Update pipeline (gene panel-additional)	OMIM: 616266	Possible (other)	PRPF40B
**58**	F	**SLC6A1**	Yes	Yes	1	Update pipeline (CNV)	-	Possible (other)	HOXD3
**143**	M	**FOXP1**	Yes	Yes	2	Update pipeline (SNV); reanalysis (additional testing)	Intronic (+ 26) loss of branchpoint; splicedefect confirmed on RNA	No cause	–
**53**	F	**ANKRD11**	No	Yes	2	Update pipeline (quality parameters)	3/7 reads (<# reads)^b^	No cause	–
**105**	M	**ANKRD11**	Yes	Yes	2	Update pipeline (quality parameters)	16/31 reads (low quality)	No cause	–
**140**	F	**TSC1**	No	Yes	1	Update pipeline (quality parameters)	21/123 (<%variant reads)^b^	No cause	–
**26**	M	**EP300**	Yes	Yes	3	Update pipeline (quality parameters)	10/78 reads (<%variant reads)	Possible (other)	ZNF41
**3**	M	**PRPS1**	No	No	1	Resequencing; variant reclassification (HGMD)	PMID: 31434166	No cause	–
**7**	F	**LAMA1**	No; yes	No	1	Resequencing	-	No cause	–
**9**	F	**ARX**	No	Yes	1	Resequencing	-	No cause	–
**24**	M	**PAK1**	No	No	3	Resequencing	-	No cause	–
**36**	F	**SATB2**	No	Yes	1	Resequencing	-	No cause	–
**79**	F	**NUS1**	No	No	3	Resequencing	-	No cause	–
**14**	F	**NSUN2**	No	No	3	Resequencing	-	Possible (other)	several
**28**	F	**KMT2B**	No	No	1	Resequencing	-	Possible (other)	PNPLA6
**47**	M	**KMT2D**	No	Yes	3	Resequencing	-	Possible (other)	ZNF711

^a^Patient ID corresponds to original publication (Vissers et al. GiM 2017) [1]

^b^Visible in BAM file

Systematic follow-up of all patients without a conclusive diagnosis after ad hoc genetic diagnostic testing (*n* = 27), and those for whom ad hoc testing was not performed (*n* = 58), revealed another 14 new conclusive genetic diagnoses; 9 resulted from bioinformatic improvements, and 5 required resequencing (Table 1—step 2 and 3, Fig. 1C, D). As the original data of 22 of the 85 patients was not compatible with current diagnostic bioinformatic pipelines (Additional file 1: Table S1), in total, 36% (37/103) of the patients in this study required resequencing for reanalysis.

**Fig. 2:**
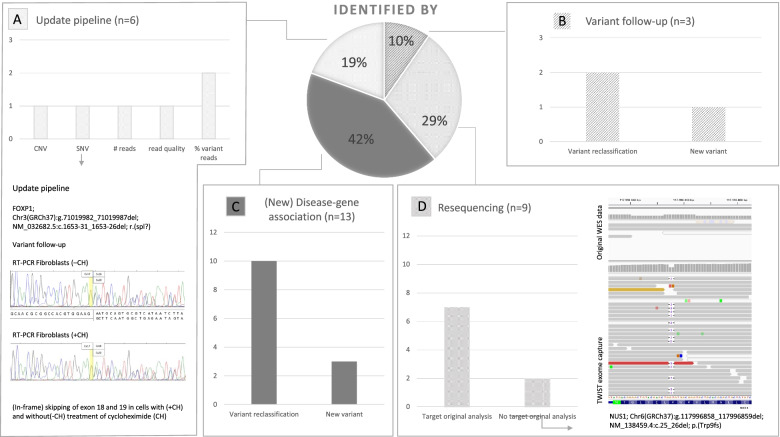
Relative contribution of changes in diagnostic analysis to increase diagnostic yield. Distribution of different reasons for finding new diagnoses in a pediatric neurology cohort. **A** Reanalysis after an update of the diagnostic pipeline was responsible for the detection of previous unrecognized copy number and (deep) intronic single nucleotide variants (CNV and SNV) and variants with too low quality criteria parameters. For instance, including interpretation of deeper intronic variants with a possible splice effect identified a variant in *FOXP1*, which after follow-up analysis was reclassified to likely pathogenic. Both (**B**) reclassification of variants based on supporting evidence from segregation analysis or metabolic investigation and (**C**) reanalysis after publication of new or broadened disease-gene associations allowed for the conclusive diagnoses of variants that were previously reported as possibly pathogenic, either in this study or in the initial WES analysis. **D** Resequencing and subsequent reanalysis identified variants that were either not targeted or not covered in the initial analysis. For instance, resequencing identified a likely pathogenic variant in *NUS1* for which the position was poorly covered in the original WES data because there was no target in the original exome capture

All analyses together elevated the total diagnostic yield in the cohort of 150 patients from 31% (*n* = 47) to 53% (*n* = 79; Fig. 1). Of the 31 novel conclusive genetic diagnoses, 12 were based on variants previously reported as possibly pathogenic [1], and 19 were based on variants that were not identified in the initial analysis (Table 1, Fig. 2).

### From VUS to conclusive diagnosis

For 10/12 previous possible diagnoses (Table 1, Fig. 2C), publications of the disease-gene associations appeared after our initial analysis of a possible diagnosis in 2015. Examples of these include *CSNK2A1* that was reported to be causative for Okur-Chung neurodevelopmental syndrome (OCNDS; OMIM #617062) in 2016 [24] and *ADPRHL2* that was associated with stress-induced childhood-onset neurodegeneration with variable ataxia and seizures (CONDSIAS; OMIM #618170) in 2018 [25]. Likewise, for one variant broadening of the phenotypes related to variants in *TBC1D24* was reported in 2019, now including rolandic epilepsy with paroxysmal exercise-induced dystonia and writer’s cramp (EPRPDC; OMIM #608105) [26]. On average, the time between the initial report of the VUS and publication of the novel disease-gene association was 3.3 years, leading to an average time to final diagnosis of 4.4 years and an average time from publication to diagnosis of 1.2 years (Additional file 2: Fig. S1). The remaining 2/12 new conclusive diagnoses were variants reclassified based on additional testing, either segregation analysis (*ACSL4*) or metabolic investigation (*HSD17B10*) (Fig. 2B).

### Conclusive diagnoses based on variants not identified in the initial analysis

For 19 patients, the conclusive genetic diagnosis was based on variants that were not detected or prioritized in the initial ES analysis (Table 1). Nine of those variants were only detected after resequencing combined with updated bioinformatic analyses and interpretation. Evaluation of the underlying explanation showed that there was no variant call in the original analysis; for 2 variants, the genomic locus was not targeted for enrichment, whereas in the other 7 cases, enrichment failed resulting in no coverage of the targeted sequence (Fig. 2D, Additional file 1: Table S1).

For 10 of 19 new diagnoses, the variants were already present in the original ES data and updates in the diagnostic pipelines allowed their detection. A failure to pass quality criteria parameters was the underlying reason in 4, such as poor coverage (*n* = 1), low quality of sequence reads (*n* = 1), and a too low percentage of variant allele frequency (*n* = 2). In a further 2 patients, enhanced CNV calling (*n* = 1) and annotation of deep(er) splice-site variants (*n* = 1) allowed for recognition of pathogenic variants that escaped attention in the initial ES analysis (Fig. 2A). At the level of variant annotation used for prioritization, updates of in silico disease gene panels acknowledged new disease-gene associations (*n* = 1) and broader phenotypic spectra of existing disease-gene association (*n* = 2) (Fig. 2C). Furthermore, in the last case, an affected sib in the family allowed for overlap analysis with the exome of the index, identifying a variant that was not prioritized by analyzing the index alone (Fig. 2B).

### Relative contribution of changes in diagnostic analysis to increase diagnostic yield

For the 31 new genetic diagnoses, we next retrospectively categorized the reasons for reaching a conclusive diagnosis. Overall, (new) disease-gene associations accounted for 42% (13/31), follow-up of variants by segregation or functional analysis accounted for 10% (3/31), reanalysis of ES data with improved diagnostic pipelines was responsible for 19% (6/31) of the additional diagnostic yield, and resequencing was essential for the last 29% (9/31) (Fig. 2).

## Discussion

Exome sequencing has been used in routine genetic testing to diagnose children with complex neurological disorders of presumed genetic origin [1, 2], with a diagnostic yield of around 30% [1, 27]. In this study, on the contribution of reanalysis of ES data, the diagnostic yield in our cohort increased from 31% to 53%. This increase of > 20% exceeds previous research reporting additional diagnostic yields of around 10–13%, with reanalysis intervals ranging between < 6 months and 3 years [10–13]. In part, this can be explained by the relative long time period of 5 years between the first and last analysis. However, more impactful, this study was not dependent on ad hoc diagnostic requests alone (17 diagnoses) but also included a systematic follow-up of the remainder of the cohort (14 diagnoses; Fig. 1). In addition, our systematic reanalysis included not only the reinterpretation of existing data, responsible for an increase of 15% (22 of 31 new genetic diagnoses), but also the generation of novel data according to the latest standard of sequencing, adding another 6% (9 diagnoses). Together, our data underscore that systematic reanalysis, in addition to ad hoc re-evaluation, can shorten the diagnostic trajectory.

### Re-evaluation and follow-up of variants of unknown significance should be standard care

In total, of the 31 novel conclusive genetic diagnoses, 14 were based on variants previously reported as possibly pathogenic, thereby contributing most to the increase of diagnostic yield. These observations are in line with increases observed in novel genotype-phenotype associations in OMIM and the expansion of phenotypes for genes with known genotype-phenotype associations as well as the number of pathogenic variants in the disease variant databases and Decipher [28–30]. Examples of reclassification of VUS in our study include a novel and distinctive phenotype for the *SRCAP* gene [31], previously associated with Floating-Harbor syndrome only, a VUS in *PRPS1* recently also reported by others [32], and small genotype-phenotype case series for *LMBRD2* [33] and *PAX3* [34] (Additional file 2: Fig. S1). Moreover, a future increase in diagnostic yield is likely to be expected as matchmaking exchange programs [35] have made it easier to establish (inter)national collaborations to generate and strengthen the disease-gene association for genes in which VUS are reported. This also underscores the need for periodic re-evaluation of VUS, as virtually at any time novel genotype-phenotype associations can be published. Also, in our study, we experienced this situation; for some patients, multiple reanalyses of the same VUS were needed before the VUS was “upgraded” to (likely) pathogenic variant. For instance, a variant in *KAT8* was found to be of unknown significance twice before its likely pathogenicity was reported [36]. Detailed analysis of all novel diagnoses based on a previous VUS in our study indicates a re-evaluation period of about 1 year for a 10–20% increase of diagnostic yield, as has been suggested by others [11, 12], and could lead to un “upgrade” up to 30% of the VUS after 2 years [28]. Importantly, re-evaluation and follow-up lead not only to “upgrading” of VUS but also to “downgrading” in some instances. In our dataset, two X-linked VUS were rejected as a possible cause after re-evaluation of the variants based on the frequency of the variant in the population.

### Diagnostic reanalysis, including resequencing, is successful and should be supported

Reanalysis focusses on existing data. However, resequencing was responsible for 29% (9/31) of the 31 novel conclusive genetic diagnoses in our study. For a few, this was because the original ES data were no longer compatible with the current bioinformatic pipelines. For the others, however, technological advances in both enrichment strategies and sequencing chemistry led to higher quality data, mostly from better and more uniform, coverage. For instance, resequencing identified a likely pathogenic variant in *NUS1* for one case. This gene was poorly covered in the original ES data and thus failed initial reanalysis variant calling algorithms (Fig. 2D). Hence, assessing improvements in technology since the original investigations can guide the decision whether one should resequence or reanalyze existing data.

### Reanalysis benefits from updates on phenotypic information

Another important factor to consider is to update clinical information before revisiting genetic data. As young patients may not (yet) display the full characteristic phenotype of a certain syndrome, reassessment of the patients’ phenotype might reveal new features implicative of specific syndromes. Such evaluations may be crucial for genetic reanalysis. Of note, such clinical reassessment may also uncover that the phenotype is not genetic in origin, but acquired, as observed for one of our patients. Secondly, assessment of the parental phenotypes is also important, as, for example, by assuming full penetrance of variants and apparently unaffected parents, variants in the index may be disregarded during interpretation [37]. It has similarly been found that incomplete penetrance or variable expressivity complicate the discovery of novel genes underlying developmental disorders [9]. This apparent nonpenetrance in clinically unaffected fathers may partly reflect under ascertainment of paternal phenotypes [38]. We observed an example of this by the identification of a duplication of 11q in an assumed to be unaffected father, whom later was known to have macrocephaly as the only feature, passing on the variant in dominant manner.

### Towards a future sustainable clinical reanalysis strategy

**Fig. 3:**
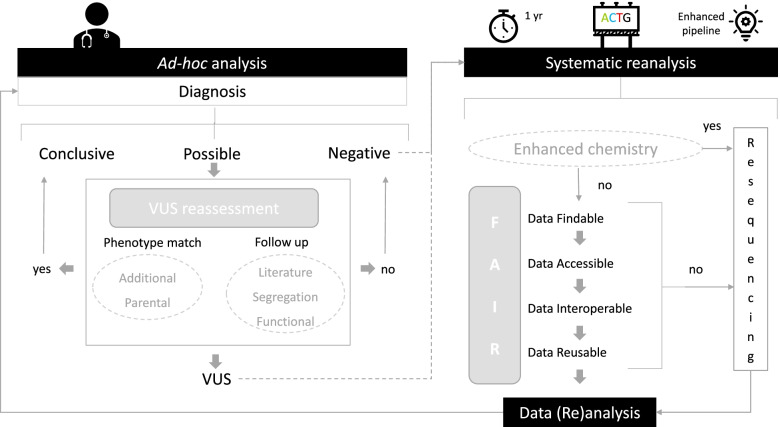
Considerations for resequencing and/or reanalysis in clinical exome sequencing. This figure depicts the considerations for each form of reanalysis, as for each individual case, it must be decided which is most suitable. Reanalysis can be initiated ad hoc or systematically based on selected time intervals or bioinformatic enhancements. Reassessment of variants of unknown significance (VUS) as well as follow-up should be performed first, using up-to-date phenotypic information and literature or additional tests for reinterpretation. When there is no conclusive diagnosis, existing data needs to be suitable for the current analysis pipeline, if not, or if state-of-the-art approaches are available, resequencing should be offered

We show that all reanalysis strategies contribute to obtaining novel diagnoses: ad hoc reanalysis upon patient/physicians request, but also systematic strategies. Thus, a combination of approaches is needed to uncover all genetic diagnoses: follow-up of VUS and reassessment of data, with or without resequencing of the samples (Fig. 3). Re-evaluation of previously reported VUS should always be performed first. Second, as diagnostic data should comply with the FAIR Guiding Principles [39] (for Findable, Accessible, Interoperable, and Reusable), existing data could be reanalyzed. Initiation of such reanalysis can either be by a (time-driven) periodic system, for instance every 1 or 2 years, or by bioinformatic enhancements, such as the implementation of analysis tools [40]. Third, we have learned that there were additional benefits from using state-of-the-art technology. It is within reason to expect that also other (future) diagnostic applications, such as (short- and long read) genome sequencing [41–43], methylation profiling [44] or optical mapping [45], will increase diagnostic yield. The feasibility of implementing such (automated) systems for reanalysis may, however, depend on available local infrastructure, bioinformatic support, and budget. It should however be noted that reanalysis can only take place with proper patient consent [46, 47]. For the ad hoc analysis, the initiative lies with the patient (who thus consents). With not all patients returning to clinic for *ad hoc* analysis, we propose to request consent from patients to allow for systematic reanalysis, also including the use of new technologies, after the initial negative diagnostic analysis to maximize benefits.

## Conclusions

We provide considerations for reanalysis of clinical exome data based on a five-year follow-up of a pediatric neurology cohort of 150 patients. The diagnostic yield in this cohort increased from 31 to 53% through a combination of ad hoc clinical and genetic diagnostic work-up and subsequent systematic reanalysis. Each reanalysis strategy, consisting of follow-up of VUS, reinterpretation of existing data, clinical reassessment of patients and parental phenotypes as well as resequencing, contributed to the additional diagnostic yield. Based on these experiences, we provide practical considerations to increase novel conclusive genetic diagnoses through reanalysis.

## Supplementary Information

**Additional file 1: Table S1.** The evolution of diagnostic yield; detailed overview of all 103 previous unsolved cases.

**Additional file 2: Fig. S1.** Timeline from first analysis, VUS discovery, publication, and diagnosis.

## Data Availability

BAM and corresponding (annotated) VCF files of patients and their parents cannot be shared under the obtained IRB approval, as patients were not consented to share raw ES data beyond the research and clinical teams. However, all variants assessed as (potentially) diagnostic relevant are presented in this paper and its supporting files. SNVs are submitted to ClinVar (https://www.ncbi.nlm.nih.gov/clinvar/; accession numbers SCV002522448-SCV002522510), the publicly available archive of human genetic variants and interpretations of their significance to disease [48]. CNVs are deposited in DECIPHER (https://www.deciphergenomics.org/; IDs 483034-483037 and 483938), a publicly available database of chromosomal imbalance and phenotype in humans [49].

## Abbreviations

CNV: Copy-number variant
ES: Exome sequencing
FAIR: Findable, Accessible, Interoperable, and Reusable
NGS: Next generation sequencing
SNV: Single-nucleotide variant
VUS: Variant of unknown significance

## References

[1] Vissers L, van Nimwegen KJM, Schieving JH, Kamsteeg EJ, Kleefstra T, Yntema HG (2017). A clinical utility study of exome sequencing versus conventional genetic testing in pediatric neurology. Genet Med.

[2] Westra D, Schouten MI, Stunnenberg BC, Kusters B, Saris CGJ, Erasmus CE (2019). Panel-based exome sequencing for neuromuscular disorders as a diagnostic service. J Neuromuscul Dis.

[3] Stark Z, Schofield D, Martyn M, Rynehart L, Shrestha R, Alam K (2019). Does genomic sequencing early in the diagnostic trajectory make a difference? A follow-up study of clinical outcomes and cost-effectiveness. Genet Med.

[4] Battaglia A, Carey JC. Diagnostic evaluation of developmental delay/mental retardation: an overview. Am J Med Genet C Semin Med Genet. 2003;117c(1):3–14. 10.1002/ajmg.c.10015. PMID: 12561053. 10.1002/ajmg.c.10015 12561053

[5] Shea SE (2006). Mental retardation in children ages 6 to 16. Semin Pediatr Neurol.

[6] Moeschler JB, Shevell M (2006). Clinical genetic evaluation of the child with mental retardation or developmental delays. Pediatrics.

[7] Romano C (2010). The clinical evaluation of patients with mental retardation/intellectual disability. Monogr Hum Genet.

[8] Vanderver A, Simons C, Helman G, Crawford J, Wolf NI, Bernard G (2016). Whole exome sequencing in patients with white matter abnormalities. Ann Neurol.

[9] Kaplanis J, Samocha KE, Wiel L, Zhang Z, Arvai KJ, Eberhardt RY (2020). Evidence for 28 genetic disorders discovered by combining healthcare and research data. Nature.

[10] Wright CF, McRae JF, Clayton S, Gallone G, Aitken S, FitzGerald TW (2018). Making new genetic diagnoses with old data: iterative reanalysis and reporting from genome-wide data in 1,133 families with developmental disorders. Genet Med.

[11] Ewans LJ, Schofield D, Shrestha R, Zhu Y, Gayevskiy V, Ying K (2018). Whole-exome sequencing reanalysis at 12 months boosts diagnosis and is cost-effective when applied early in Mendelian disorders. Genet Med.

[12] Li J, Gao K, Yan H, Xiangwei W, Liu N, Wang T (2019). Reanalysis of whole exome sequencing data in patients with epilepsy and intellectual disability/mental retardation. Gene.

[13] Wenger AM, Guturu H, Bernstein JA, Bejerano G (2017). Systematic reanalysis of clinical exome data yields additional diagnoses: implications for providers. Genet Med.

[14] Tan NB, Stapleton R, Stark Z, Delatycki MB, Yeung A, Hunter MF (2020). Evaluating systematic reanalysis of clinical genomic data in rare disease from single center experience and literature review. Mol Genet Genomic Med.

[15] Basel-Salmon L, Orenstein N, Markus-Bustani K, Ruhrman-Shahar N, Kilim Y, Magal N (2019). Improved diagnostics by exome sequencing following raw data reevaluation by clinical geneticists involved in the medical care of the individuals tested. Genet Med.

[16] Ji J, Leung ML, Baker S, Deignan JL, Santani A (2021). Clinical exome reanalysis: current practice and beyond. Mol Diagn Ther.

[17] Deignan JL, Chung WK, Kearney HM, Monaghan KG, Rehder CW, Chao EC (2019). on behalf of the ALQA. Points to consider in the reevaluation and reanalysis of genomictest results: a statement of the American College of Medical Genetics and Genomics(ACMG). Genet Med.

[18] Pfundt R, Del Rosario M, Vissers L, Kwint MP, Janssen IM, de Leeuw N (2017). Detection of clinically relevant copy-number variants by exome sequencing in a large cohort of genetic disorders. Genet Med.

[19] Lelieveld SH, Reijnders MRF, Pfundt R, Yntema HG, Kamsteeg E-J, de Vries P (2016). Meta-analysis of 2,104 trios provides support for 10 new genes for intellectual disability. Nat Neurosci.

[20] McLaren W, Gil L, Hunt SE, Riat HS, Ritchie GR, Thormann A (2016). The Ensembl Variant Effect Predictor. Genome Biol.

[21] Karczewski KJ, Francioli LC, Tiao G, Cummings BB, Alföldi J, Wang Q (2020). The mutational constraint spectrum quantified from variation in 141,456 humans. Nature.

[22] Practice guidelines for the evaluation of pathogenicity and the reporting of sequence variants in clinical molecular genetics; 2013. https://www.acgs.uk.com/media/10791/evaluation_and_reporting_of_sequence_variants_bpgs_june_2013_-_finalpdf.pdf.

[23] Silva M, de Leeuw N, Mann K, Schuring-Blom H, Morgan S, Giardino D (2019). European guidelines for constitutional cytogenomic analysis. Eur J Hum Genet.

[24] Okur V, Cho MT, Henderson L, Retterer K, Schneider M, Sattler S (2016). De novo mutations in CSNK2A1 are associated with neurodevelopmental abnormalities and dysmorphic features. Hum Genet.

[25] Ghosh SG, Becker K, Huang H, Dixon-Salazar T, Chai G, Salpietro V (2018). Biallelic mutations in ADPRHL2, encoding ADP-ribosylhydrolase 3, lead to a degenerative pediatric stress-induced epileptic ataxia syndrome. Am J Hum Genet.

[26] Lüthy K, Mei D, Fischer B, De Fusco M, Swerts J, Paesmans J (2019). TBC1D24-TLDc-related epilepsy exercise-induced dystonia: rescue by antioxidants in a disease model. Brain.

[27] Eldomery MK, Coban-Akdemir Z, Harel T, Rosenfeld JA, Gambin T, Stray-Pedersen A (2017). Lessons learned from additional research analyses of unsolved clinical exome cases. Genome Med.

[28] Salinas V, Vega P, Marsili L, Pérez-Maturo J, Martínez N, Zavala L, et al. The odyssey of complex neurogenetic disorders: From undetermined to positive. Am J Med Genet C Semin Med Genet. 2020. 10.1002/ajmg.c.31848. PMID: 33084218. 10.1002/ajmg.c.31848 33084218

[29] Köhler S, Gargano M, Matentzoglu N, Carmody LC, Lewis-Smith D, Vasilevsky NA (2021). The human phenotype ontology in 2021. Nucleic Acids Res.

[30] Bragin E, Chatzimichali EA, Wright CF, Hurles ME, Firth HV, Bevan AP, Swaminathan GJ. DECIPHER: database for the interpretation of phenotype-linked plausibly pathogenic sequence and copy-number variation. Nucleic Acids Res. 2014;42(Database issue):D993–D1000. 10.1093/nar/gkt937. PMID: 24150940. 10.1093/nar/gkt937 PMC3965078 24150940

[31] Rots D, Chater-Diehl E, Dingemans AJM, Goodman SJ, Siu MT, Cytrynbaum C (2021). Truncating SRCAP variants outside the Floating-Harbor syndrome locus cause a distinct neurodevelopmental disorder with a specific DNA methylation signature. Am J Hum Genet.

[32] Meng L, Wang K, Lv H, Wang Z, Zhang W, Yuan Y (2019). A novel mutation in PRPS1 causes X-linked Charcot-Marie-Tooth disease-5. Neuropathology.

[33] Malhotra A, Ziegler A, Shu L, Perrier R, Amlie-Wolf L, Wohler E (2021). De novo missense variants in LMBRD2 are associated with developmental and motor delays, brain structure abnormalities and dysmorphic features. J Med Genet.

[34] Hart J, Miriyala K (2017). Neural tube defects in Waardenburg syndrome: a case report and review of the literature. Am J Med Genet A.

[35] Sobreira N, Schiettecatte F, Valle D, Hamosh A (2015). GeneMatcher: a matching tool for connecting investigators with an interest in the same gene. Hum Mutat.

[36] Li L, Ghorbani M, Weisz-Hubshman M, Rousseau J, Thiffault I, Schnur RE (2020). Lysine acetyltransferase 8 is involved in cerebral development and syndromic intellectual disability. J Clin Invest.

[37] Salfati EL, Spencer EG, Topol SE, Muse ED, Rueda M, Lucas JR (2019). Re-analysis of whole-exome sequencing data uncovers novel diagnostic variants and improves molecular diagnostic yields for sudden death and idiopathic diseases. Genome Med.

[38] Wright CF, Eberhardt RY, Constantinou P, Hurles ME, FitzPatrick DR, Firth HV (2021). Evaluating variants classified as pathogenic in ClinVar in the DDD Study. Genet Med.

[39] Wilkinson MD, Dumontier M, Aalbersberg IJ, Appleton G, Axton M, Baak A (2016). The FAIR Guiding Principles for scientific data management and stewardship. Scientific Data.

[40] van der Sanden B, Corominas J, de Groot M, Pennings M, Meijer RPP, Verbeek N, et al. Systematic analysis of short tandem repeats in 38,095 exomes provides an additional diagnostic yield. Genet Med. 2021. 10.1038/s41436-021-01174-1. PMID: 33846582. 10.1038/s41436-021-01174-1 33846582

[41] Helman G, Lajoie BR, Crawford J, Takanohashi A, Walkiewicz M, Dolzhenko E (2020). Genome sequencing in persistently unsolved white matter disorders. Ann Clin Transl Neurol.

[42] Vanderver A, Bernard G, Helman G, Sherbini O, Boeck R, Cohn J (2020). Randomized clinical trial of first-line genome sequencing in pediatric white matter disorders. Ann Neurol.

[43] Pauper M, Kucuk E, Wenger AM, Chakraborty S, Baybayan P, Kwint M (2021). Long-read trio sequencing of individuals with unsolved intellectual disability. Eur J Hum Genet.

[44] Sadikovic B, Levi MA, Kerkhof J, Aref-Eshghi E, Schenkel L, Stuart A, et al. Clinical epigenomics: genome-wide DNA methylation analysis for the diagnosis of Mendelian disorders. Genet Med. 2021. 10.1038/s41436-020-01096-4. PMID: 33547396. 10.1038/s41436-020-01096-4 PMC8187150 33547396

[45] Chaisson MJP, Sanders AD, Zhao X, Malhotra A, Porubsky D, Rausch T (2019). Multi-platform discovery of haplotype-resolved structural variation in human genomes. Nat Commun.

[46] Giesbertz NAA, van Harten WH, Bredenoord AL (2019). A duty to recontact in genetics: context matters. Nat Rev Genet.

[47] Mitchell C, Ploem C, Retèl V, Gevers S, Hennekam R (2020). Experts reflecting on the duty to recontact patients and research participants; why professionals should take the lead in developing guidelines. Eur J Med Genet.

[48] Landrum MJ, Lee JM, Benson M, Brown GR, Chao C, Chitipiralla S (2018). ClinVar: improving access to variant interpretations and supporting evidence. Nucleic Acids Res.

[49] Firth HV, Richards SM, Bevan AP, Clayton S, Corpas M, Rajan D (2009). DECIPHER: database of chromosomal imbalance and phenotype in humans using Ensembl resources. Am J Hum Genet.

