# Supplementary material for: Reanalysis of exome negative patients with rare disease: a pragmatic workflow for diagnostic applications
Source: Genome Med. 2022 Jun 17;14:66. doi: 10.1186/s13073-022-01069-z (PMC9204949; doi:10.1186/s13073-022-01069-z)
Supplement: Supplementary file 2 — Additional file 2: Fig. S1. Timeline from first analysis, VUS discovery, publication, and diagnosis. [file 13073_2022_1069_MOESM2_ESM.pptx]

## Slide 1
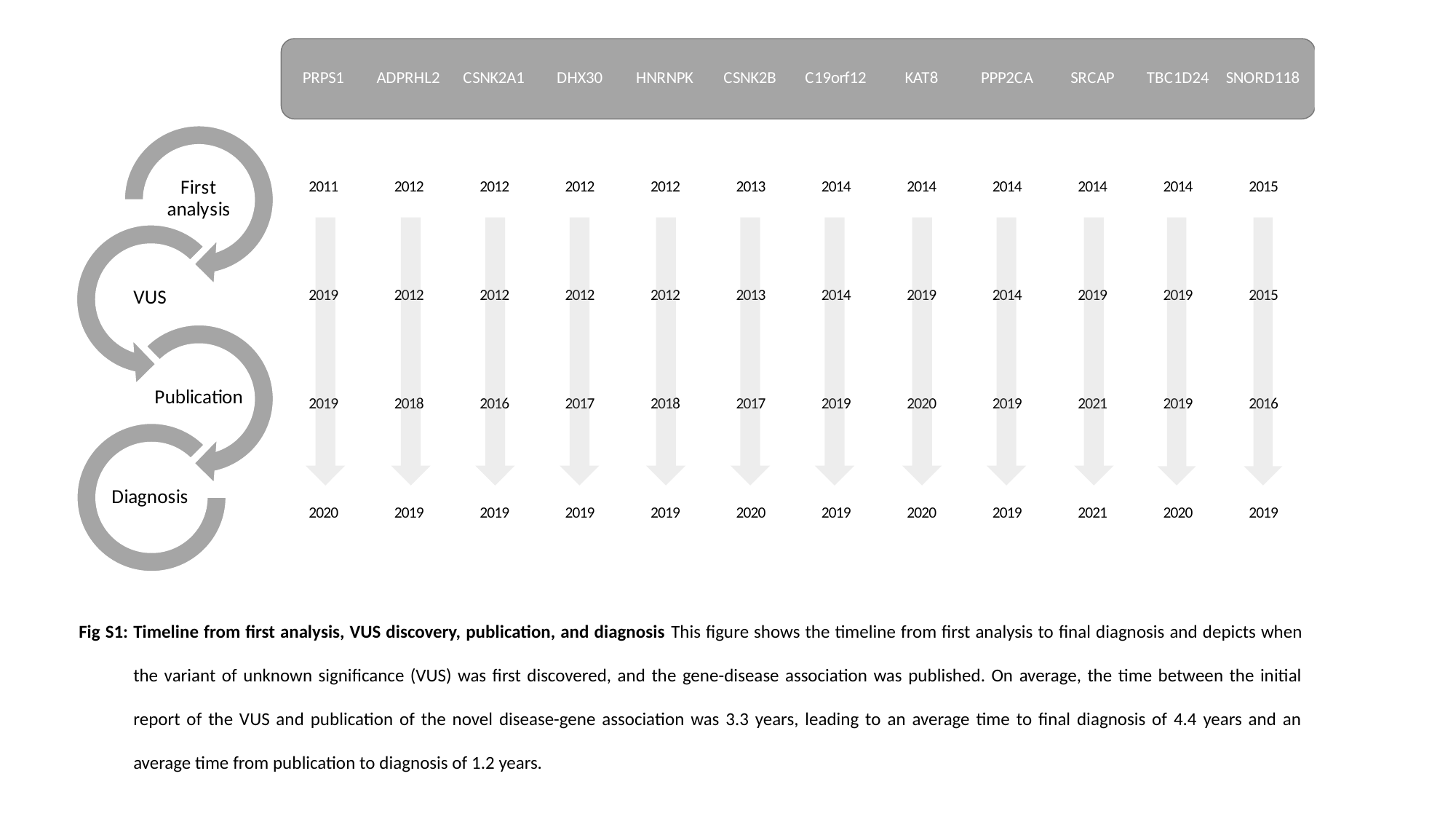

Fig S1: Timeline from first analysis, VUS discovery, publication, and diagnosis This figure shows the timeline from first analysis to final diagnosis and depicts when the variant of unknown significance (VUS) was first discovered, and the gene-disease association was published. On average, the time between the initial report of the VUS and publication of the novel disease-gene association was 3.3 years, leading to an average time to final diagnosis of 4.4 years and an average time from publication to diagnosis of 1.2 years.
